# A computational analysis of SARS cysteine proteinase-octapeptide substrate interaction: implication for structure and active site binding mechanism

**DOI:** 10.1186/1471-2105-10-S1-S48

**Published:** 2009-01-30

**Authors:** Krongsakda Phakthanakanok, Khanok Ratanakhanokchai, Khin Lay Kyu, Pornthep Sompornpisut, Aaron Watts, Surapong Pinitglang

**Affiliations:** 1 Division of Biochemical Technology, School of Bioresources and Technology, King Mongkut's University of Technology Thonburi, Bangkok, Thailand; 2 Computational Chemistry Unit Cell, Department of Chemistry, Faculty of Science, Chulalongkorn University, Bangkok, Thailand; 3 School of Biosciences, The University of Exeter, Exeter, UK; 4 Department of Food Science and Technology, School of Science, University of the Thai Chamber of Commerce, Bangkok, Thailand; 5 INTO University of Exeter, The Old Library, Exeter, UK

## Abstract

**Background:**

SARS coronavirus main proteinase (SARS CoVMpro) is an important enzyme for the replication of Severe Acute Respiratory Syndrome virus. The active site region of SARS CoVMpro is divided into 8 subsites. Understanding the binding mode of SARS CoVMpro with a specific substrate is useful and contributes to structural-based drug design. The purpose of this research is to investigate the binding mode between the SARS CoVMpro and two octapeptides, especially in the region of the S3 subsite, through a molecular docking and molecular dynamics (MD) simulation approach.

**Results:**

The one turn α-helix chain (residues 47–54) of the SARS CoVMpro was directly involved in the induced-fit model of the enzyme-substrate complex. The S3 subsite of the enzyme had a negatively charged region due to the presence of Glu47. During MD simulations, Glu47 of the enzyme was shown to play a key role in electrostatic bonding with the P3Lys of the octapeptide.

**Conclusion:**

MD simulations were carried out on the SARS CoVMpro-octapeptide complex. The hypothesis proposed that Glu47 of SARS CoVMpro is an important residue in the S3 subsite and is involved in binding with P3Lys of the octapeptide.

## Background

The human coronavirus is a major cause of respiratory syndrome and in particular of a disease called Severe Acute Respiratory Syndrome (SARS). This disease spread rapidly from China to several countries during 2003. The enzyme called Severe Acute Respiratory Syndrome Coronavirus Main Proteinase (SARS CoVMpro) is an important enzyme involved in the life cycle of the human coronavirus. The crystal structure of the SARS CoVMpro complexed to the inhibitor has been solved previously [1-5]. This enzyme is a member of the cysteine proteinases and exhibits the typical catalytic diad, cysteine and histidine, in the active site [6-12]. Previous studies on substrate specificity indicated that the active site, including the binding site, of the SARS CoVMpro could bind specifically to a substrate containing 8 amino acids. These positions in the octapeptide were termed P5-P4-P3-P2-P1-P1'-P2'-P3' and the sequence Ser-Ala-Val-Leu-Gln-Ser-Gly-Phe. This sequence is optimal for the proteinase from Transmissible Gastro Enteritis Virus (TGEV), but it is unsuitable for SARS CoVMpro [7]. Recently, investigations of substrate specificity against SARS CoVMpro proposed that the octapeptides with sequences of Ser-Ala-Val-Leu-Gln-Ala-Gly-Phe and Thr-Val-Lys-Leu-Gln-Ser-Gly-Phe are optimal for cleavage by SARS CoVMpro. It is interesting to note that when the P3 position of this latter octapeptide was changed from Val to Lys it caused an increase in the rate of catalysis (*k*_cat_/*K*_m_) of the SARS CoVMpro of 4.31 fold [13]. However, information on interactions between P3Lys of the octapeptide and the S3 subsite of the SARS CoVMpro remain unclear. Therefore, our research intends to study the interactions between P3Lys and the S3 subsite in order to investigate the amino acids in the S3 subsite that are critical for binding P3Lys of the octapeptide. In addition, the two octapeptides Thr-Val-Arg-Leu-Gln-Ala-Gly-Phe and Thr-Val-Ile-Leu-Gln-Ala-Gly-Phe were used to investigate these interactions. At the P3 position these octapeptides contained either a long chain positive charge or an aliphatic hydrophobic amino acid. This was proposed to prove that the P3Lys is a significant amino acid for binding in the S3 subsite of the SARS CoVMpro. Thus, molecular modeling techniques can be used to clarify this problem [14,15]. In the present paper, we present the 2 ns conventional MD simulation of the SARS CoVMpro complexed with the octapeptide and compare this to an uncomplexed SARS CoVMpro, with the purpose of investigating the amino acids in each subsite, and especially S3, which are crucial to increasing the substrate binding. Hence, the results obtained from this research are very important in the understanding of the binding mechanism and catalytic mechanism of the SARS CoVMpro.

## Methods

### Visualization and computational

All steps in this research were performed *in silico *using molecular modeling software. Preparation of all three-dimensional structures used Insight II (version 2001) from Accelrys [16]. Molecular docking and molecular dynamics (MD) simulations used Autodock 3.0.5, AutodockTools and GROMACS 3.3.1 [17-19]. The results of molecular docking and MD simulations were analyzed using Discovery Studio 2.0.1 [16]. All of the calculations were performed on a 48 processor Itanium cluster at the National Electronic and Computer Technology Center (NECTEC), Thailand.

### Preparation the structures of the SARS CoVMpro and the octapeptide

The structure of the SARS CoVMpro was taken from the Brookhaven protein data bank using the PDB code 1UK4[10]. The structure was checked and the missing atoms replaced using the Builder module of the Insight II. The polar hydrogen atoms were added followed by energy minimization *in vacuo *with steepest descent method for 1,000 steps. The final structure obtained from energy minimization was used in all further steps. The structures of the octapeptides were prepared based on the research of Fan et al. [13]. Three octapeptides were constructed, including hydrogen atoms, by using the Builder module and the following sequences: Thr-Val-Lys-Leu-Gln-Ala-Gly-Phe, Thr-Val-Arg-Leu-Gln-Ala-Gly-Phe and Thr-Val-Ile-Leu-Gln-Ala-Gly-Phe. These structures only differed at the P3 position. All of the structures were subjected to energy minimization in the same manner as that for SARS CoVMpro. The structures obtained from energy minimization were utilized for further docking studies.

### Structure refinement

For this research, the structure of the SARS CoVMpro was prepared in the catalytically competent conformation. It is believed that this structure was in the natural form and ready for substrate catalysis [14,15]. Firstly, the structure of the SARS CoVMpro, obtained from the energy minimization, was subjected to primary MD simulation. To perform MD simulation the structure of the enzyme was set to the GROMOS96 43a1 force field with explicit hydrogen atoms in the aromatic rings. The simulation cell was created in a cubic periodic box with a minimum distance of 0.9 Å between the protein and the box walls. The enzyme was soaked with approximately 40,000 water molecules defined using the simple point charges (SPC) of water model. The Glu and Asp of the SARS CoVMpro were set a charge of -1. Lys was set a charge of +1. His had an added hydrogen atom at the B position. As the total charge of the system was -6, six atoms of Na^+ ^were added to the system to adjust the charge to neutral. Electrostatic interactions between charged groups at a distance of less than 3 Å were calculated explicitly. Energy minimization was performed by using 1,000 steps of steepest descent method. Long-range electrostatic interactions were calculated using the Particle-Mesh Ewald method (PME) with a grid width of 1.2 Å and a fourth-order spline interpolation. A cutoff distance of 9 Å was applied for Lennard-Jones interactions. To maintain the system at a constant temperature of 300 K, a Berendsen thermostat was applied using a coupling time of 0.1 ps. Pressure was held at 1 bar, with a coupling time of 1 ps. The time step was set as 2 fs and the simulation performed for 100 ps. The structure of the SARS CoVMpro obtained at the last time point (100 ps) was selected for further molecular docking studies.

### Molecular docking studies

In the molecular docking, the interaction between the SARS CoVMpro and the three octapeptides were investigated. The structure of the enzyme obtained from the final step of structure refinement and the structures of the ocapeptide obtained from energy minimization were used in all steps. The calculations employed a Lamarckian Genetic Algorithm (LGA) with partial flexible rotatable bonds. The structure of the octapeptide was defined as 19 rotatable bonds, situated along the backbone, by fixing the position of the side chains. The grid points for Autogrid calculations were set to be 80 × 80 × 80 Å with the sulfur atom of SARS CoVMpro Cys145 assigned as the center of the grid box. The docking parameters were set to a LGA calculation of 1,000 runs. The energy evaluations were set to 1,500,000 and 27.000 generations. Population size was set to 100 and the rate of gene mutation and the rate of gene crossover were set to 0.02 and 0.8 respectively. At the end of the calculations, the obtained conformations were summarized, collected and extracted using the AutodockTool. The docked conformations were clustered by set the Root Mean Square Deviation (RMSD) as 2.0 Å. The first ranked presented the highest members was selected. The conformation that exhibited the lowest docking energy in that cluster was selected for analysis and further MD simulation.

### Molecular dynamics simulation

In order to perform molecular dynamics simulations, three structures of the SARS CoVMpro complexed with the octapeptides obtained from the docking, were prepared. The parameters of the simulation were adjusted to be the same as in the structure refinement step described above. However, one atom of the Na+ was also added in each system of the enzyme-substrate complexed of the octapeptide P3Lys and P3Arg due to it neutralized the amino acid, Lys and Arg. In the simulation, each time step was set to 2 fs and the simulation of the whole system performed for 2,000 ps (2 ns). The structure of the enzyme-substrate complexes were extracted every 1 ps for analysis.

### Data analysis

The results of simulations with each structure were compared against each other. Values of the root mean square deviation (RMSD) and the root mean square fluctuation (RMSF) were monitored during the simulations. These values were the criteria used to describe the motion of the protein chains, including the amino acids, in the active site of the SARS CoVMpro. The atomic distances and number of hydrogen bonds were also investigated to explain the interaction between the SARS CoVMpro and the octapeptide in the enzyme-substrate complex.

## Results and discussion

### Catalytically competent conformation of the SARS CoVMpro

**Figure 1:**
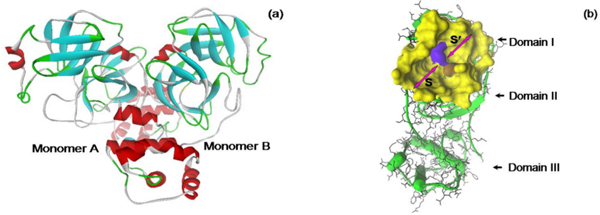
**Three dimensional structure of the SARS CoVMpro**. The structures were shown in dimer form (a) and monomer form (b). The structure was extracted at the equilibrium point of MD simulation. The active site is shown in yellow surface and it is separated into the S and S' sites.

The active site region of the SARS CoVMpro was composed of 7 protein chains: chain 1, residues 19–28; chain 2, residues 41–54; chain 3, residues 67–70; chain 4, residues 117–120; chain 5, residues 139–145; chain 6, residues 163–172 and chain7, residues 187–192. Among them, chain 2 was called the one turn α-helix and chain 7 was called the long loop. Previous research has determined the equilibrium point of the system of the SARS CoVMpro free form, and found that the enzyme reached equilibrium after 400 ps [14,15]. Therefore, initially in this research, MD simulation of the dimer structure of the SARS CoVMpro free form was performed for 500 ps (Figure 1). Then, the structure of the enzyme was snapshot and the new confirmation compared to the starting structure. It had a different conformation in the active site caused by movement of the one turn α-helix and the long loop chain of the enzyme. The RMSD value was calculated from this comparison and was 2.62 Å. This result indicated that the structure of the SARS CoVMpro and, in particular, the active site obtained from this research differed from the crystal structure. However, this new structure was considered an acceptable conformation for further docking and MD simulation, since the orientation of the protein chains in the active site were consistent with previously reported research [7,15,20,21]. During simulation, the protein one turn α-helix and long loop chain stretched their backbone. This movement resulted in an "open mouth" catalytic cleft between domain I and domain II [15]. This phenomenon is the catalytically competent conformation of the SARS CoVMpro and this structure was used for further docking and MD simulation.

### Docking studies

The active site of the SARS CoVMpro was divided into 8 subsites. Thus, the subsites namely S5-S4-S3-S2-S1-S1'-S2'-S3' accommodated the corresponding peptide residues named P3-P2-P1-P1'-P2'-P3', respectively. It is known that the S2 subsite is a hydrophobic pocket and it is located near the catalytic dyad between domains I and II. The S1 subsite is a deep hydrophilic pocket and is located close to Cys145, in part of domain II [7].

The docking energy of the three octapeptides was calculated. The conformation with the lowest docking energy from the best cluster (highest of each substrate member) was selected. The docking energy was a criterion that was used to judge specificity of the substrate. This meant that, the lower docking energy referred to a higher specificity. For comparison, the energies obtained from docking of each octapeptide are listed in Table 1, showing the octapeptide P3Lys had an energy of -14.23 kcal/mol. It had the lowest docking energy so it was the most specific. Below these were P3Arg and P3Ile, which had docking energies of -13.26 and -8.11 kcal/mol, respectively. Hence, the octapeptide P3Ile had the lowest specificity.

**Figure 2:**
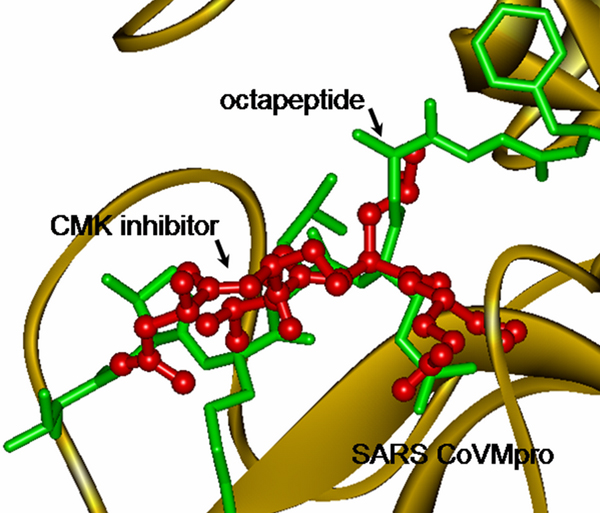
**Molecular docking of the octapeptide substrate**. The docking performed through superimposition between the hexapeptide chloromethyl ketone inhibitor (red ball and stick) and the octapeptide (green stick).

The model of the docked structure, as shown in Figure 2, indicated that the orientation of all of the octapeptides were similar. Therefore, the decrease in the docking energy was due to the potential differences in the P3 position of the octapeptide. It was suggested that an electrostatic interaction of the Lysine, attracted to a nearby Glutamic Acid, was the cause. However, the S3 position of the SARS CoVMpro is composed of two Glutamic Acids (Glu47 and Glu166). To date, it is not known which Glutamic Acid interacts with P3Lys. Therefore, this research investigated this problem by studying the interactions of the enzyme substrate complex through MD simulations. In addition, the orientation of the docking octapeptides was superimposable over the peptidomimic chloromethyl ketone inhibitor. This inhibitor was specifically bound to the SARS CoVMpro in the crystal structure (PDB ID = 1UK4). Therefore, the structure of the enzyme-substrate complex obtained from docking studies was acceptable for further MD simulations.

**Table 1:** The docking energy. The energies obtained from the interactions between the SARS CoVMpro and the octapeptide substrates.

**Rank**	**Name**	**Sequence**^a^	**Docking energy (kcal/mol)**
1	P3Lys	TVKLQAGF	-14.23

2	P3Arg	TVRLQAGF	-13.26

3	P3Ile	TVILQAGF	-8.11

^a ^Mutated residues are underlined

### MD simulations of the interactions between the SARS CoVMpro and the octapeptide in form of enzyme-substrate complexed

**Figure 3:**
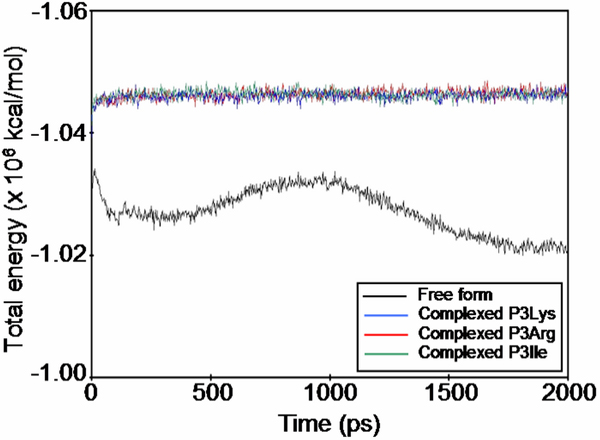
**Time dependence of the total energy from MD simulation**. The lines of the SARS CoVMpro free form, complexed with octapeptide P3Lys, P3Arg and P3Ile were colored in black, blue, red and green, respectively.

The structure of the enzyme-substrate complex (SARS CoVMpro-octapeptide) obtained from molecular docking was then subjected to MD simulation. During simulation, the total energy, the RMSD and the RMSF of the system were monitored. The total energy is an important criterion to investigate the equilibrium point and the values obtained for each system are shown in Figure 3. It is clear that the system of the enzyme in complex with the octapeptide rapidly reached equilibrium.

The equilibrium point of this system was about 100 ps. The average value of the total energy was determined between 100 and 200 ps. This showed that the energy of the complex system with octapeptide P3Lys, P3Arg and P3Ile were -104521, -104482 and -104216 kcal/mol, respectively. These results showed that the complex of octapeptide P3Lys had the lowest energy, whereas P3Ile had the highest energy. In contrast to the enzyme free form, the results of the total energy value was unstable in the first 1500 ps. The values changed distinctly in the first 100 ps and then reached a stable level up to 500 ps. After this time, the values were changed again by first increasing and then decreasing until 1500 ps. However, the values reached an equilibrium level at nearly 2000 ps. From these results, we have suggested that the variation of the total energy in the enzyme free form is caused by movement of the flexible chain, one turn α-helix and long loop, in the active site of the SARS CoVMpro. On the other hand, in the active site of the SARS CoVMpro complex, the octapeptide contributed to maintaining the conformation of the enzyme. To examine this further, the motion of the octapeptides was also investigated.

**Figure 4:**
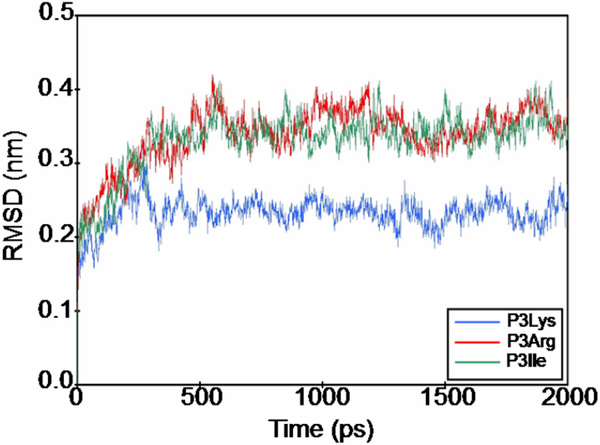
**Time dependence of the RMSD from MD simulation**. The lines of the SARS CoVMpro free form, complexed with octapeptide P3Lys, P3Arg and P3Ile were colored in black, blue, red and green, respectively.

The RMSD values versus simulation time are shown in Figure 4. The RMSD values of all octapeptides were dramatically increased in the first 100 ps. After this time, the values of P3Lys stabilized, whereas the values of the P3Arg and P3Ile continued to increase and reached a stable level after nearly 500 ps. After 500 ps, all of values were stable until the end of simulation. In the stable period, the mean of the RMSD values of P3Lys, P3Arg and P3Ile were 0.23, 0.33 and 0.34, respectively. These values indicated that, the structures of the octapeptide in the stable period had moved slightly when compared to the starting structure.

It is possible to interpret from the results that the variability in the first 100 ps was due to the structure of the octapeptides adapting, after which the P3Lys was a close fit to the active site cleft of the SARS CoVMpro. After this time, the octapeptide P3Arg and P3Ile were still not in the correct confirmation for the active site cleft and they moved about 0.3–0.35 nm away. However, it is also possible to suggest that the movement was only happening on some of the residues of the octapeptide, because the RMSD values stabilized after 500 ps. This indicated that some residues of P3Arg and P3Ile remained in the active site cleft.

### Movement investigation of the amino acids involved with the interactions between the SARS CoVMpro and the octapeptide

**Figure 5:**
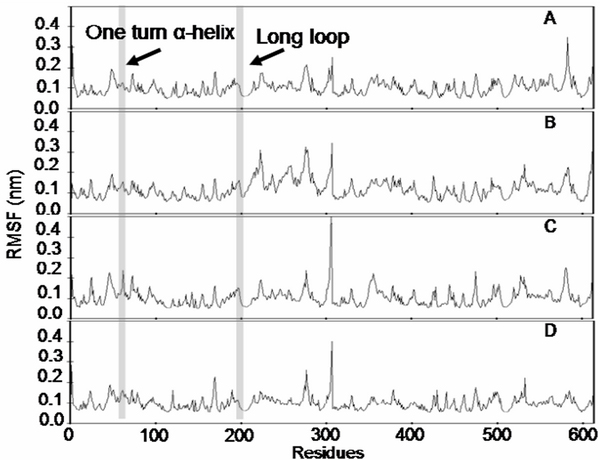
**The RMSF with respect to amino acid residues of the SARS CoVMpro**. The values were calculated averaging at time range 500–2000 ps of MD simulation. The values of the SARS CoVMpro complexed with octapeptide P3Lys, P3Arg and P3Ile were shown in A, B and C, respectively. The value of the SARS CoVMpro free form was shown in D. The shadow region from left to right represent the region of protein chain namely one turn α-helix (residues 47–54) and long loop (residues 184–192) of SARS CoVMpro, respectively.

The RMSF of the enzyme and the octapeptide has also been investigated. This criterion could describe which residues were either moving or fixed during simulation. The values were calculated by averaging movement in the equilibrium period from a time of 500–2000 ps of simulation. The results shown in Figure 5 demonstrate that the fluctuation of the amino acids in the active site (highlight of the amino acid residues number) of the enzyme complexed with P3Lys (Figure 5A), were lower than with the other octapeptide complexes or the free form. The RMSF in the enzyme complexed with P3Arg also had low values, but these were still greater than those seen for P3Lys (Figure 5B). This is in contrast to the RMSF values in complex with P3Ile (Figure 5C) which they still remain high values. However, the value of the long loop chain (right highlight) is seen to be consistent for all structures. The results also indicated that in the enzyme free form (Figure 5D) the one turn α-helix moved during simulation, which is in contrast to the enzyme complexed with octapeptide P3Lys, where the one turn α-helix chain maintained its position. From the results of MD simulations, it was found that the octapeptides in which P3 is either Lys or Arg have not fallen out of the active site of SARS CoVMpro during simulation. At the first time period of MD simulations, these P3 have their side chains pointed out to solvent and that after the observed conformational changes these would then point upward to Glu47, and not downward to Glu166. The results also found that, at equilibration of MD simulations, an electrostatic interaction was formed between S3Glu47 and P3Lys or P3Arg with a distance of 3.5–5.0 Å.

We found the Glu166 could not generate the electrostatic interaction with the octapeptide P3 because it was neutralized with a salt bridge to Ser1 of monomer B. However, The P3Lys was bound more specifically than P3Arg due to the steric hindrance effect of the planar Arg side chain. The hydrogen bonds, water-bridges and electrostatic interactions between S3Glu47 and P3Lys (Arg) have been observed continuously until the end of MD simulation times. This phenomenon has not been previously reported and this research can propose that the characteristic of S3Glu47, with the negatively charged region it generates, is important for the binding mechanism of SARS CoVMpro.

### The hydrogen bonds of the enzyme-substrate complex

**Figure 6:**
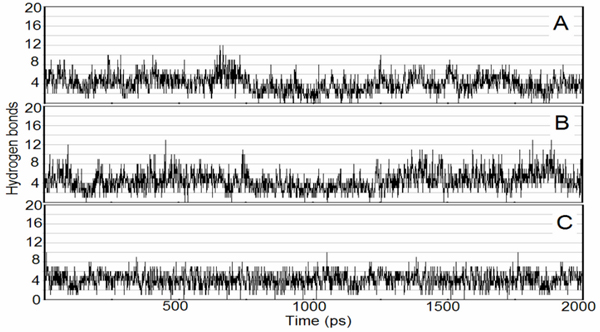
**Hydrogen bonds number between the SARS CoVMpro and the octapeptide**. The octapeptide P3Lys, P3Arg and P3Ile were shown in A, B and C, respectively.

The number of hydrogen bonds seen within the simulation time fluctuates between 4–7 (Figure 6), which indicated that there were always 4–7 hydrogen bonds between the SARS CoVMpro and the octapeptide. However, the enzyme-substrate complexed could form 9 hydrogen bonds at P5Thr(N-terminus)---S5Gln191, P4Val(backbone)---S5Thr190, P4Val(backbone)---Thr191, P3Lys---S3Glu47, P1Gln---S1His163, P1Gln---Ser144, P3'Phe---S4Thr21, P3'Phe---S4Thr24 and P3'Phe---S4Thr26. These bonds may be involved in ensuring effective binging of the octapeptide to the active site of the SARS CoVMpro.

## Conclusion

The MD simulation results and the number of hydrogen bonds formed in the SARS CoVMpro-octapeptide complex have been obtained. These results indicate clearly that the S3 subsite of the SARS CoVMpro has a negative character. The hypothesis proposed that Glu47 is an important residue in the S3 subsite for binding with P3Lys of the octapeptide. The electrostatic interactions between Glu47 and P3Lys play a key role in specific binding. These observations are very important and provide further information for structural-based drug design against SARS virus.

## List of abbreviations used

GROMACS: Groningen Machine for Chemical Simulations; LGA: LamarckianGenetic Algorithm; MD: Molecular Dynamics; PDB: Protein Data Bank; PME: Particle-Mesh Ewald; RMSD: Root Mean Square Deviation; RMSF: Root Mean Square Fluctuation; SARS CoVMpro: SARS Coronavirus MainProteinase; SPC: Simple Point Charges; TGEV: Transmissible GastroEnteritis Virus; VMD: Visual Molecular Dynamics.

## Competing interests

The authors declare that they have no competing interests.

## Authors' contributions

KP designed the calculation plan, carried out the results and drafted the manuscript. KR and KLK designed the biological problem to be solved, analyzed the results and provided the computer machines. PS helped to draft the manuscript and interpreted the result of MD simulations. AW helped to draft, suggested the methodology and improved English in the manuscript. SP helped to interpret the results, conceived of and supervised the study in addition to assisting with the drafting of the manuscript. All authors read and approved the final manuscript.
